# Chemical and pharmacological investigation of micropropagated *Hygrophila pogonocalyx* produced from leaf explants

**DOI:** 10.1186/1999-3110-54-51

**Published:** 2013-10-30

**Authors:** Chin-Wen Ho, Rong-Dih Lin, Tzong-Huei Lee, Chien-Hung Lin, Chi-Luan Wen, Yu-Ting Tseng, Mei-Hsien Lee

**Affiliations:** 1 Department of Bioengineering, Tatung University, Taipei, 104 Taiwan; 2 Department of Internal Medicine, Ho-Ping Branch, Taipei City Hospital, Taipei, 100 Taiwan; 3 Graduate Institute of Pharmacognosy, College of Pharmacy, Taipei Medical University, Taipei, 110 Taiwan; 4 Seed Improvement and Propagation Station, Council of Agriculture, Taichung, 515 Taiwan; 5 Center for Reproductive Medicine & Sciences, Taipei Medical University Hospital, Taipei, 110 Taiwan

**Keywords:** Anti-melanogenesis, Constituents, *Hygrophila pogonocalyx*, Indirect shoot organogenesis from leaf explants, Neurocytoprotection

## Abstract

**Background:**

An optimized method for indirect shoot organogenesis from the leaf explants of *Hygrophila pogonocalyx*, a rare and endemic species in Taiwan, was developed to supply enough quantity of plant materials for the first chemical and pharmacological investigation.

**Results:**

Incubation of the young leaves on Murashige and Skoog (MS) medium supplemented with 6-benzylaminopurine (0.5 mg/l) and indole-3-acetic acid (0.1 mg/l) resulted in the best multiplication rate for organogenesis. The average number of adventitious buds per leaf was 22.8 ± 1.9 after 8-week culture. The adventitious buds rooted and developed into plantlets when cultured simply on MS medium. Using this protocol, up to 37,600 plants were produced from a single leaf explant in one year. From the ethanol extract of the leaves of this micropropagated plant, 13 compounds were isolated and identified, including two flavones (**1**, **11**), four flavonols (**9**, **10**, **12**, and **13**), three phenylethanoid glycosides (**6**–**8**), two alkylated glycosides (**2**–**3**), and two steroids (**4**–**5**). Of these, acteoside (**7**) exhibited anti-tyrosinase activity in human epidermal melanocytes and luteolin 7-*O*-β-d-glucopyranoside (**11**) exhibited the greatest neurocytoprotective activity.

**Conclusions:**

The method, indirect shoot organogenesis from leaf explants of *H. pogonocalyx*, could be developed to supply enough quantity of plant materials for the chemical and pharmacological investigation. In the present study, the isolated active compounds may develop for whitening agents or treating neurodegenerative diseases in the future.

**Electronic supplementary material:**

The online version of this article (doi:10.1186/1999-3110-54-51) contains supplementary material, which is available to authorized users.

## Background

The extracts from plants of the *Hygrophila* genus (Acanthaceae) have been demonstrated to possess anti-tumor (Mazumdar et al. 1997), anti-bacterial (Khan and Omoloso 2002), hepatoprotective (Raj et al. 2010; Shanmugasundaram and Venkataraman 2006), free radical scavenging, anti-lipid peroxidation activities (Shanmugasundaram and Venkataraman 2006; Vijayakumar et al. 2006), and inhibit gentamicin-induced nephrotoxicity (Bibu et al. 2010). *H. auriculata* was reported to exhibit significant anti-diabetic activity in addition to potent antioxidant activity in diabetic individuals (Vijayakumar et al. 2006) and *H. difformis* exhibited significant protective activity against strychnine- and leptazol-induced convulsions (Pal and Samanta 2011).

*Hygrophila pogonocalyx* Hayata (Acanthaceae), a perennial aquatic water plant, is an endemic species in Taiwan (Hsieh and Huang 1974). Plant tissue culture techniques offer a viable tool for the mass multiplication of identical plant material and the germplasm conservation of rare endangered plants. These techniques can provide a continuous supply of plant materials from elite germplasm lines, which can help exploit the therapeutic properties of these plant species and eliminate the need for harvesting specimens from the wild. Thomas and Yoichiro (2010) standardized an *in vitro* propagation protocol for the rare medicinal plant *Justicia gendarussa* using nodal explants, and this improved method for plant regeneration is helpful for the study of phytochemical production (Balaraju et al. 2008; Sudha and Seeni 1994). Balaraju et al. reported an efficient regeneration protocol for a valuable medicinal plant, *Vitex agnus-castus*, and all regenerated plants exhibited high homogeneity (Vijayakumar et al. 2006).

In a previous study, tissue culture and plant regeneration *via* direct shoot organogenesis induced from the shoot tip or axially bud of *H. pogonocalyx* was reported (Huang and Win 1999). However, indirect shoot organogenesis from leaf explants has never been reported in this species. In our previous study, the 95% ethanol (EtOH) extract of *H. pogonocalyx* exhibited free radical scavenging activities (Jiang et al. 2006). Therefore, the objective of this research was to develop a simple and highly efficient regeneration protocol using leaf explants and examine the antioxidant activities of the regenerated plants. The compounds from regenerated plants of *H. pogonocalyx* were also isolated, and their structures and activities were evaluated.

## Methods

### Plant material

*Hygrophila pogonocalyx* Hayata (600 g) was collected from the Highlands Experiment Farm, National Taiwan University, Taiwan and identified by Mr. Chi-Luan Wen, Seed Improvement and Propagation Station, Council of Agriculture, Taiwan. A voucher specimen (M-380) was deposited at the Graduate Institute of Pharmacognosy (Taipei Medical University, Taipei, Taiwan).

### Shoot proliferation and plant regeneration

For shoot organogenesis, young leaves were used as explants and cultured on Murashige and Skoog (Murashige and Skoog 1962) basal medium supplemented with BA, NAA, IAA, or 2iP at different concentrations, as shown in Table 1. The media were supplemented with 3% (w/v) sucrose and solidified with 0.7% (w/v) agar, and the pH was adjusted to 5.7. The adventitious buds rooted and regenerated into plantlets when cultured on MS medium without plant regulators. For the mass production of plantlet, six to eight node explants cut from a regenerated plantlet were cultured in sterile vessels with 100 ml of liquid MS medium supplemented with 3% (w/v) sucrose. The rooted plantlets were transplanted to a potting mixture (1:1, peat moss: vermiculite) with garden soil. The potted plants were acclimatized for 4 weeks and then transferred to the field.

**Table 1:** **Effect of plant growth regulators on callus induction and shoot regeneration of leaf explants of** ***Hygrophila pogonocalyx*** **Hayata**

Plant growth regulators (mg/l)	4-week-culture Callus induction (%)	8-week culture	
		Shoot no./explant	Mean stem length (cm)
BA (0.1) + NAA (0.1)	100	6.2 ± 0.8^d^*	1.8 ± 0.2^a^
BA (0.5) + NAA (0.1)	100	12.2 ± 1.9^b^	0.8 ± 0.1^d^
BA (1.0) + NAA (0.1)	100	3.8 ± 1.3^e^	0.7 ± 0.1^d^
BA (0.1) + IAA (0.1)	100	6.6 ± 1.1^d^	1.4 ± 0.2^b^
BA (0.5) + IAA (0.1)	100	22.8 ± 1.9^a^	1.0 ± 0.1^c^
BA (1.0) + IAA (0.1)	100	9.2 ± 1.3^c^	0.8 ± 0.1^d^
BA (0.1) + IAA (0.1) + 2iP (1)	100	7.2 ± 0.8^d^	1.7 ± 0.2^a^
BA (0.5) + IAA (0.1) + 2iP (1)	100	11.4 ± 1.1^b^	1.0 ± 0.2^c^
BA (1.0) + IAA (0.1) + 2iP (1)	100	9.0 ± 0.7^c^	0.7 ± 0.1^d^

*: In a single column, means with the same letter are not significantly different according to Duncan’s multiple range tests at the 0.05 level.

### Extract preparation

The aerial parts of plants were harvested monthly, frozen at −80°C for 24 h, and lyophilized for 48 h. All lyophilized samples were stored at room temperature.

### General experimental procedures

Column chromatography was performed using Diaion HP 20P (100–200 mesh, Mitsubishi Chemical Industries, Tokyo, Japan), Sephadex LH-20 (100 μm; Pharmacia Fine Chemicals, Piscataway, NJ), MCI gel CHP 20P (Supelco, Bellefonte, PA, USA), and octadecyl silane (ODS) columns (Merck, Darmstadt, Germany). TLC was performed on pre-coated Si gel 60 F254 plates (Merck). The ^1^H and ^13^C NMR spectra were recorded on an Avance DRX 500 instrument (Bruker Madison, WI). Electrospray ionization-mass spectrometry spectra were obtained on a VG platform electrospray mass spectrometer (VG Analytical, Ipswich, UK).

### Extraction and isolation

The leaves of the regenerated *H. pogonocalyx* (3 kg) were macerated with 95% EtOH at room temperature for 5 days, then filtered to give the residue and filtrate. The residue was treated in a similar manner as above three times. The combined filtrates were concentrated under reduced pressure to give the EtOH extract (186 g), which was divided into fractions soluble in *n*-hexane, ethyl acetate (EtOAc) and H_2_O by liquid-liquid partitioning. The EtOAc extract (30 g) was re-suspended in H_2_O, subjected to chromatography on a Diaion HP-20 column, eluted with MeOH − H_2_O (0%, 20%, 40%, 60%, and 100%) and analyzed by thin layer chromatography to obtain seven respective fractions (E-1–7). Fractions E-3 and E-4 were passed through a Sephadex LH-20 column (95% EtOH) to obtain 13 (E-3-1–13) and 11 (E-4-1–11) subfractions, respectively. Re-crystallization of fraction E-3-11 (20 mg) with MeOH yielded compound (**1**) (12 mg). Fraction E-4-3 (130 mg) was separated by semi-preparative HPLC (Biosil 5 ODS-W column, 10 × 250 mm; solvent system: 50% MeOH; flow rate: 3.0 ml/min; detector: 254 nm) to give compounds (**2**) (15 mg) and (**3**) (16 mg). Fraction E-4-5 (45 mg) was separated by semi-preparative HPLC (solvent system: 20–100% MeOH in 60 min) to obtain compounds (**4**) and (**5**) (6 mg). Fraction E-4-6 (40 mg) was separated by semi-preparative HPLC (solvent system: 25% acetonitrile and 35% MeOH, respectively) to give compound (**6**) (4 mg). Compounds (**7**) (29 mg) and (**8**) (5 mg) were obtained from fraction E-4-7 (120 mg) by semi-preparative HPLC (solvent system: 25% acetonitrile and 40% MeOH). Compound (**9**) (5 mg) was obtained from fraction E-4-9 (43 mg) by semi-preparative HPLC (solvent system: 30% MeOH). Fraction E-6 (100 mg) was subjected to an ODS column and eluted with 20–100% MeOH to obtain compound (**10**) (7 mg).

The *n*-butanol extract (35 g) was eluted on a Sephadex LH-20 column with 100% MeOH to obtain nine fractions (B-1–9). After monitoring by HPLC evaluation, B-6 was subjected to MCI gel CHP 20P column chromatography. Fraction B-6 was eluted with a stepwise gradient of aqueous methanol (H_2_O to 100% MeOH), yielding 14 fractions (B-6-1–14). A precipitate was evident in the B-6-5 fraction (85 mg). Re-crystallizing the precipitate with MeOH and H_2_O yielded pure compound (**11**) (21 mg). Compound (**12**) (10 mg) was obtained from B-6-14 (120 mg). A precipitate from B-9 (105 mg) was re-crystallized with MeOH and H_2_O to yield pure compound (**13**) (11 mg). The spectral data and physical constants for isolated compounds were included in Supporting information (Additional file 1).

### Antioxidant activities

#### 1, 1-Diphenyl-2-picrylhydrazyl (DPPH) radical scavenging activity

DPPH radical scavenging effect was measured according to the method of Hou et al. (Hou et al. 2003). Each tested sample was mixed with 160 μM DPPH in an MeOH solution. After a 20-min incubation at room temperature in the dark, the absorbance was read at 517 nm. The inhibitory percentage of DPPH was calculated according to the following equation:

$$\begin{array}{l} \text{DPPH}\;\text{radical}\;\text{scavenging}\;\text{activity}\;\left(\%\right)\\ \;=\left[(A0-A1)/A0\right]\times 100\%. \end{array}$$

A0 was the absorbance of the control (blank, without extract), and A1 was the absorbance in the presence of the tested samples.

#### Ferrous ion chelating activity

The ferrous ion chelating activity was determined by the Fe^2+^-ferrozine test system using the method of Erdogan-Orhan et al. (Erdogan-Orhan et al. 2010). In brief, the test samples were incubated with 2 mM FeCl_2_ solution. The reaction was initiated by adding ferrozine solution to the mixture and incubating the mixture for 10 min at room temperature. The absorbance of the reaction mixture was measured at 562 nm. The ratio of inhibition of ferrozine-Fe^2+^ complex formation was calculated as follows:

$$\%\mathrm{Inhibition}=\left[(A0-A1)/A0\right]\times 100\%.$$

A0 was the absorbance of the control (blank, without extract), and A1 was the absorbance in the presence of the tested samples.

#### Total phenol

The amount of total phenolics in the extracts was determined according to the method of Hou et al. (Hou et al. 2003). The test sample solution was mixed with the Folin-Ciocalteu reagent, 20% sodium carbonate (Na_2_CO_3_) solution, and water. After incubation for 25 min at room temperature, the reaction mixture was centrifuged at 5000 rpm for 10 min. The absorbance of the supernatant was measured at 730 nm by using a spectrophotometer. The amount of total phenolics was expressed as gallic acid equivalents in milligrams per gram dry plant extract.

### Anti-melanogenic activity

#### Cell viability of human epidermal melanocytes (HEMn cells)

Cells (1 × 10^5^) were added to individual wells of a 24-well plate. After incubation for 24 h, a test sample (100 μM) was added to each well and incubated for another 24 h. Cell viability was then determined at 450 nm on a μQuant microplate reader (Bio-Tek Instruments, Inc.) by using the WST-8 cell proliferation assay.

#### Cellular tyrosinase activity in HEMn cells

Cellular tyrosinase activity was measured using a previously described method (Lee et al. 2006). After treatment with individual compounds (100 μM) for 24 h, the cells were washed with potassium phosphate-buffered saline (PBS) and lysed with PBS (pH 6.8) containing 1% Triton X-100. Protein content was determined using a commercial protein assay kit (Bio-Rad, Hercules, CA). After quantifying protein levels, 40 μg of protein, 2.5 mM l-DOPA, and 0.1 M PBS (pH 6.8) was added to each well (the same protein content) of a 96-well plate. After incubation at 37°C for 1 h, the absorbance was measured at 475 nm by using an enzyme-linked immunosorbent assay reader.

### Neurocytoprotective activity

#### PC12 cell culture

PC12 cells (2 × 10^5^) were grown in RPMI 1640 medium supplemented with horse serum (10%) and fetal bovine serum (5%) at 37°C in a humidified 5% CO_2_ atmosphere (Lin et al. 2010). Cells were seeded in the plate and cultured with 100 ng/ml nerve growth factor (NGF) for 5 days. 6-Hydroxydopamine (6-OHDA) was used to produce oxidative stress. PC12 cells were treated with the test samples (100 μM) for 6 h before exposure to 175 μM 6-OHDA (Lin et al. 2010).

#### Cell viability and neurocytoprotective activity of PC12 cells

PC12 cell growth was evaluated using the WST-8 assay (Lin et al. 2010). PC12 cells were seeded on a 96-well plate in culture medium and NGF for 5 days and then treated with the test compounds (100 μM) for 24 h. WST-8 reagent was added, and cells were incubated for 4 h, after which their viability was analyzed using a μQuant microplate reader (Bio-Tek Instruments, Winooski, VT, USA) at 450 nm. The absorbance values of the experimental cultures were used to indicate the levels of cell viability. Neurocytoprotective activity was evaluated the cell viability that the differentiation PC12 pre-treated with the test compounds (100 μM) for 6 h before exposure to 6-OHDA.

#### Statistical analysis

Experiments were replicated three times for each analysis, and data were analyzed by analysis of variance (ANOVA) using Statistical Analysis system (SAS) and tested for significance by Duncan’s multiple range test (Duncan 1955) at the 5% level.

## Results

### Effect of plant growth regulators on shoot organogenesis from leaf explants

**Figure 1:**
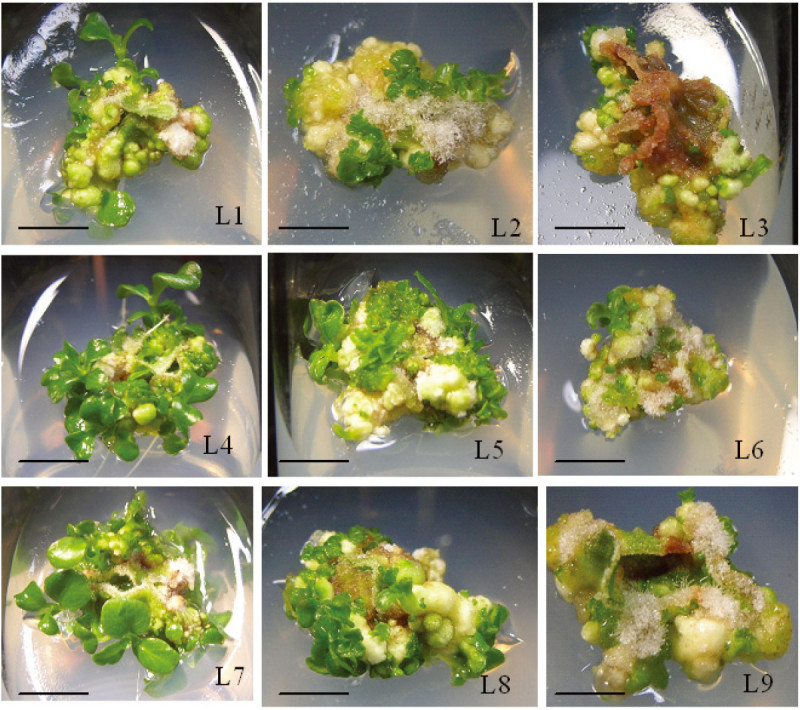
**Callus and adventitious shoot regeneration of leaf explants of** ***Hygrophila pogonocalyx*** **cultured with different concentrations (mg/l) of plant growth regulators. L1**: BA (0.1) + NAA (0.1); **L2**: BA (0.5) + NAA (0.1); **L3**: BA (1) + NAA (0.1); **L4**: BA (0.1) + IAA (0.1); **L5**: BA (0.5) + IAA (0.1); **L6**: BA (1) + IAA (0.1); **L7**: BA (0.1) + IAA (0.1) + 2iP (1) ; **L8**: BA (0.5) + IAA (0.1) + 2iP(1); **L9**: BA (1) + IAA (0.1) + 2iP (1) (bar = 1 cm).

Friable callus developed from leaf explants, after which adventitious buds were visible on the surface of the callus (Figure 1). The callus induction rate was 100% when leaf explants were cultured on nine test media. Table 1 shows the effect of different concentrations of plant growth regulators on the average number of shoots produced per explant and average shoot length. The MS basal medium supplemented with 0.5 mg/l BA and 0.1 mg/l IAA provided the best multiplication rate, with an average value of 22.8 ± 1.9 buds per explant after 8 weeks of culture. The average shoot length was higher when the medium contained a low BA concentration (0.1 mg/l) in combination with IAA, NAA, or 2iP.

### Plant regeneration

**Figure 2:**
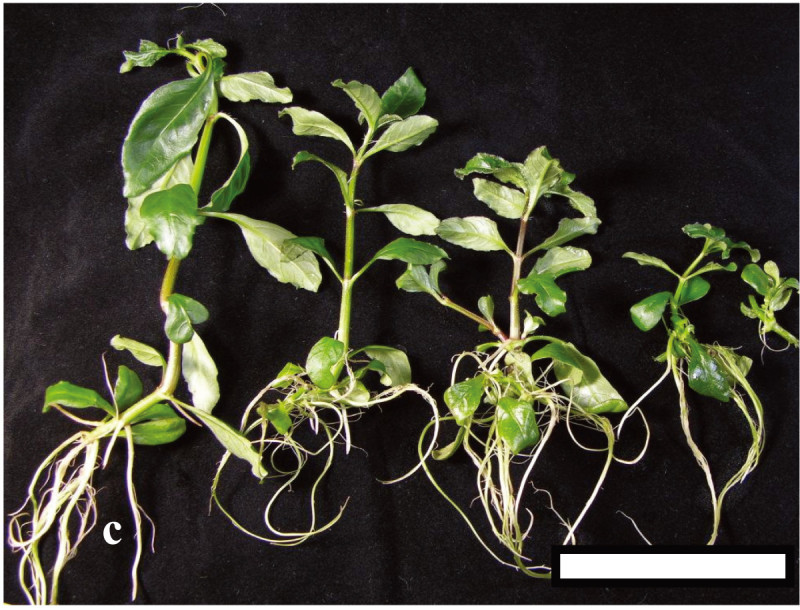
**The adventitious buds rooted and regenerated into plantlets when cultured on MS medium without plant regulators.** Bar = 7.5 cm.

The adventitious buds rooted and regenerated into plantlets when cultured on MS medium without plant regulators after 1 week. For the mass production of plantlets, six to eight node explants were cultured in sterile vessels with liquid MS medium. After 6 weeks of culture, the rooted plantlets (Figure 2) were transplanted to a potting mixture (1:1, peat moss: vermiculite) with garden soil. The potted plants were acclimatized for 4 weeks and then transferred to the field. In 1 year, using the above protocol, 37,600 plants could be produced from a single leaf explant.

### Antioxidant activities

The antioxidant activities of tissues of *H. pogonocalyx* collected in different seasons are presented in Table 2. Tissues harvested in June exhibited the highest DPPH radical scavenging activity (3175.4 ± 17.8 mg AA/100 g DW) and total phenol content (127.1 ± 0.0 mg GA/100 g DW). The ferrous ion chelating activity of tissues collected in May was substantial (57.0 ± 13.3 μmol EDTA/g DW).

**Table 2:** **The effect of season on the antioxidant activities of the ethanol extracts of** ***Hygrophila pogonocalyx*** **Hayata**

	Ferrous ion chelating activity (%)	DPPH radical scavenging activity	Total phenol
	μmol EDTA/g DW*	AEAC**	mg GA/100 g DW^#^
March	8.3 ± 0.0 ^c, ##^	2914.0 ± 25.0 ^bc^	117.5 ± 0.0 ^c^
April	7.4 ± 0.0 ^c^	2495.5 ± 7.8 ^d^	91.6 ± 0.0 ^f^
May	57.0 ± 13.3 ^a^	2727.4 ± 9.2 ^cd^	98.6 ± 0.0 ^e^
June	17.5 ± 1.2 ^b^	3175.4 ± 17.8 ^a^	127.1 ± 0.0 ^a^
September	4.1 ± 0.2 ^c^	2979.6 ± 356.8 ^ab^	122.3 ± 0.0 ^b^
October	4.4 ± 0.1 ^c^	2542.2 ± 43.3 ^d^	92.3 ± 0.0 ^f^
November	3.3 ± 0.2 ^c^	2619.8 ± 24.4 ^d^	106.0 ± 0.0 ^d^
December	2.8 ± 0.1 ^c^	2896.9 ± 36.6 ^bc^	118.8 ± 0.1 ^bc^

*: μmol EDTA/g DW: micromole EDTA equivalents per gram of plant dry weight.

**: Ascorbic acid equivalent antioxidant activity: milligram of ascorbic acid equivalents per 100 g of plant dry weight (mg AA/100 g DW).

#: mg GA/100 g DW: milligrams of gallic acid equivalents per 100 g of plant dry weight.

##: In a single column, means with the same letter are not significantly different according to Duncan’s multiple range tests at the 0.05 level.

### Purification and identification of constituents of tissues produced by indirect shoot organogenesis from leaf explants of *H. pogonocalyx*

**Figure 3:**
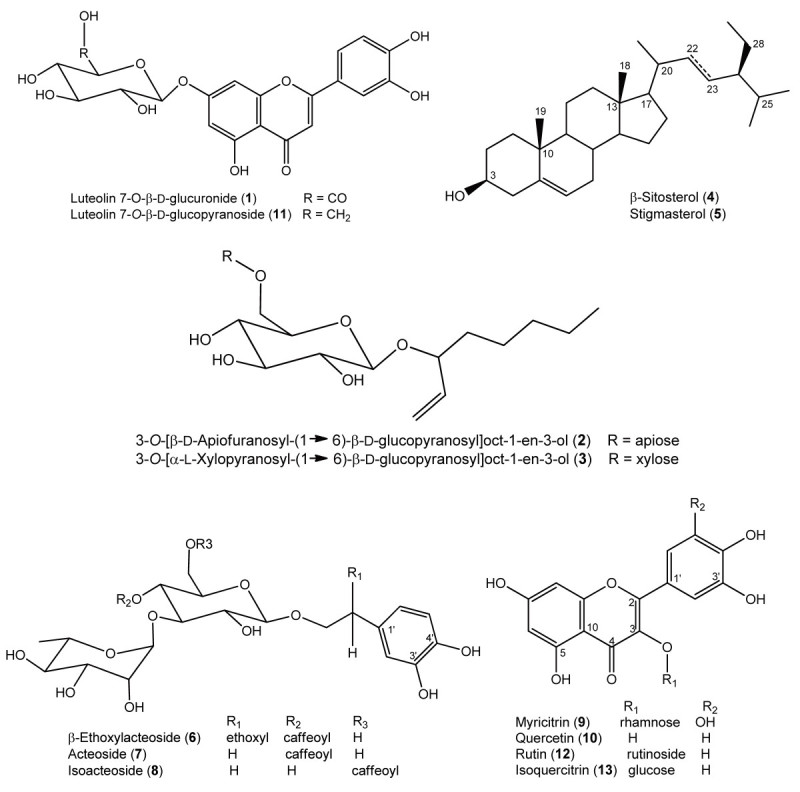
**Structures of the isolated compounds from leaf explants of** ***H. pogonocalyx*** **.**

In the present study, the leaves produced by indirect shoot organogenesis from leaf explants of *H. pogonocalyx* were extracted with 95% EtOH, and then phytochemical investigations were conducted. The extract was re-suspended in H_2_O and partitioned with *n*-hexane, ethyl acetate, and *n*-butanol sequentially. HPLC-directed isolation (of the EtOAc and *n*-butanol fractions) was performed after subjecting these fractions to Diaion HP-20, Sephadex LH-20, and MCI CHP-20P column chromatography and semi-HPLC purification. From the EtOAc and *n*-butanol fractions, we obtained 10 ((**1**)-(**10**)) and 3 compounds ((**11**)-(**13**)), respectively. Structural identification of these compounds was achieved by comparison of their physical data (^1^H and ^13^C NMR, MS) spectral data with those reported in literature. They belong to flavones (luteolin 7-*O*-β-d-glucuronide (**1**) (Lee et al. 2002) and luteolin 7-*O*- β-d-glucopyranoside (**11**) (Shi et al. 2008)), flavonols (myricetin (**9**) (Ibrahim et al. 2001), quercetin (**10**) (Min et al. 2010), rutin (**12**) (Zou et al. 2010), and isoquercitrin (**13**)), phenylethanoid glycosides (β-ethoxylacteoside (**6**) (Jun et al. 2003), acteoside (**7**) (Henry et al. 1987), and isoacteoside (**8**) (Kim et al. 2001)), alkylated glycosides (3-*O*-[β-d-apiofuranosyl-(1 → 6)-β-d-glucopyranosyl]oct-1-en-3-ol (**2**) (Zou et al. 2008) and 3-*O*-[α-l-xylopyranosyl-(1 → 6)-β-d-glucopyranosyl]oct-1-en-3-ol (**3**) (Yamamura et al. 1998), and steroids (β-sitosterol (**4**) and stigmasterol (**5**) (Hisash et al. 1990)). The structures of these compounds are shown in Figure 3. These compounds represent substances isolated from leaves produced by indirect shoot organogenesis from leaf explants of *H. pogonocalyx* for the first time. Excluding the steroids (**4**) and (**5**), each of the isolated constituents was examined separately at a relatively high concentration (100 μM) for anti-melanogenic and neurocytoprotective activities.

### Cytotoxicity and anti-melanogenic activity of isolated constituents from *H. pogonocalyx* in HEMn cells

**Figure 4:**
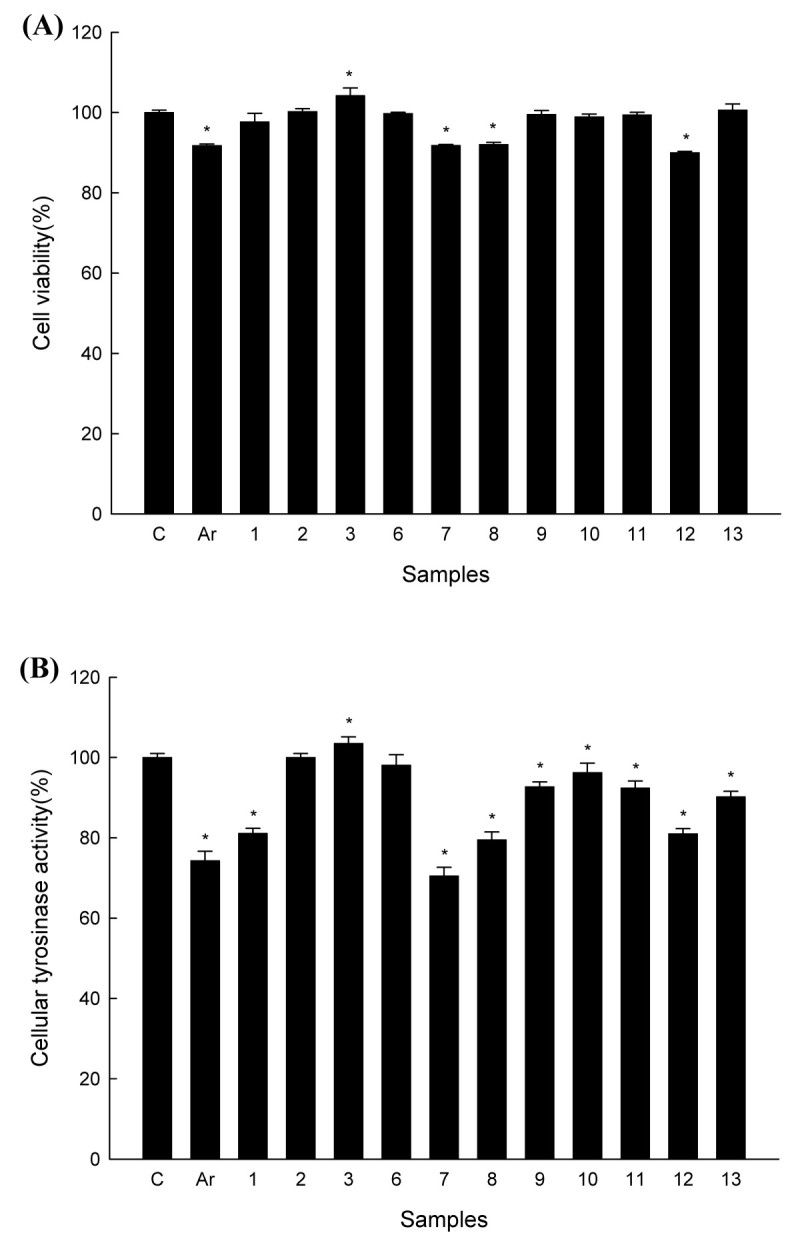
**Cytotoxicity and cellular anti-tyrosinase activity of the isolated constituents of** ***H. pogonocalyx*** **in human epidermal melanocytes (HEMn cells).** HEMn cells (1 × 10^5^) were treated with the positive control arbutin (Ar) and the isolated compounds (100 μM) for 24 h. Afterward, **(A)** the supernatant was removed and incubated with WST-8 cell counting reagent for 4 h at 37°C. The absorbance was measured using a microplate reader at 450 nm. **(B)** The lysates (equal amount of proteins) were incubated with l-dopa at a final concentration 2.5 mM for 1 h at 37°C. Each determination was made in triplicate, and the data shown represent means ± SD. *P-value < 0.05 when compared with control.

The isolated constituents from *H. pogonocalyx* were further evaluated for anti-melanogenic activity. Using the MTT assay, cells were exposed to 11 test samples, and all cells exhibited greater than 85% viability (Figure 4A) after a 24-h treatment, demonstrating that the isolated compounds exhibited no or little cytotoxicity in HEMn cells. Afterward, the 11 test compounds were then examined for cellular anti-tyrosinase activity. Acteoside (**7**) exhibited greater anti-tyrosinase activity than the positive control arbutin, and luteolin 7-*O*-β-d-glucopyranoside (**1**), isoacteoside (**8**), and rutin (**12**) displayed anti-tyrosinase activity (Figure 4B).

### Cytotoxicity and neurocytoprotective activity of isolated constituents from *H. pogonocalyx* in NGF-differentiated PC12 cells

The NGF-differentiated PC12 cells were used as a model to assess neurocytoprotective activity in the present study. In our previous study, we found that when NGF-differentiated PC12 cells were treated with 175 μM 6-OHDA for 24 h, cell viability decreased to 50.0±4.6% compared with that of the untreated cells (Lin et al. 2009). Therefore, we used 175 μM 6-OHDA to induce cytotoxicity in the subsequent experiment.

**Figure 5:**
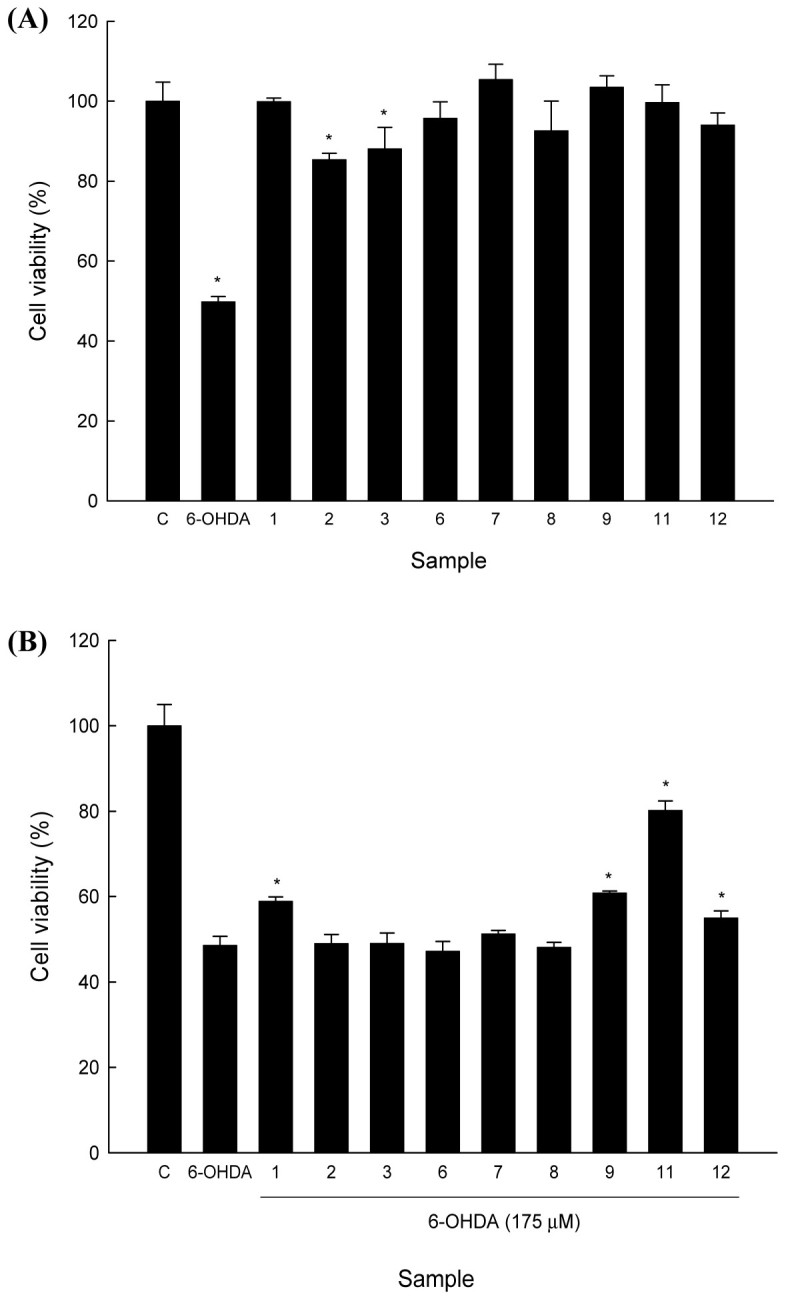
**Cytotoxicity and neurocytoprotective activity of the isolated constituents of** ***H. pogonocalyx*** **in 6-OHDA-induced NGF-differentiated PC12 cells. (A)** NGF-differentiated PC12 cells (2 × 10^5^) were treated with 6-OHDA (175 μM) and tested samples (100 μM) for 24 h. **(B)** The NGF-differentiated PC12 cells (2 × 10^5^) were treated with the tested constituents (100 μM) for 6 h and then were treated with 6-OHDA (175 μM) for 24 h. The cell-protective activity was calculated as follows: (OD_450_ of the sample/OD_450_ of 6-OHDA) × 100. Each determination was made in triplicate, and the data shown represent means ± SD. *P-value < 0.05 when compared with control.

Using the WST-8 assay to evaluate cytotoxicity, PC12 cells were exposed to the test samples, and all cells exhibited greater than 90% viability following 24 h of treatment, which indicated that the isolated compounds did not induce PC12 cell cytotoxicity (Figure 5A). The NGF-differentiated PC12 cells were incubated with the test compounds (100 μM) prior to 6-OHDA exposure, and luteolin 7-*O*-β-d-glucopyranoside (**11**) exhibited potent neurocytoprotective activity. Luteolin 7-O-β-d-glucuronide (**1**), myricetin (**9**), and rutin (**12**) exhibited slightly protective activities (Figure 5B).

## Discussion

*H. pogonocalyx* is a rare endemic species in Taiwan. The extract of *H. pogonocalyx* exhibited the free radical scavenging activities in our previous study (Jiang et al. 2006). Plant tissue culture is often used for plant propagation. Different techniques in plant tissue culture may offer the ability to generate exact copies of plants. Using this technique, the proliferation of a large number of specific plant tissues or cells can be controlled in an external environment to create a regeneration system to produce a large population of seedlings and then achieve the conservation of the sources of plant species. In the present report, we develop a simple and highly efficient regeneration protocol using leaf explants. The percentage of callus induction in leaf explant of *H. pogonocalyx* was 100% on MS medium supplemented with all tested plant growth regulators and combinations (Table 1). Explants cultured on medium containing 0.5 mg/l BA combined with NAA, IAA, or 2iP exhibited effective shoot regeneration from callus. The highest number of shoots produced per explant was 22.8 ± 1.9. The longest shoots were produced from leaf explants (1.8 ± 0.2 cm) cultured on medium containing 0.1 mg/l BA supplemented with NAA, IAA, or 2iP. In the present investigation, BA played an important role as a plant growth regulator, and it had a significant effect on the average number of shoots per explant. Similar findings were obtained for *Justicia gendarussa* using nodal explants, as the maximal shoot induction was obtained on MS medium supplemented with 17.7 μM BA (Balaraju et al. 2008 Sudha and Seeni 1994), and for the micropropagation of *V. agnus-castus* from nodal and meristem explants, the highest shoot regeneration was produced using MS medium supplemented with 2 mg/l BA (Balaraju et al. 2008; Sudha and Seeni 1994).

Rooting occurred with regenerated shoots cultured on MS medium without plant growth regulators. However, 9.8 μM IBA mostly effectively induced rooting (73%) in *J. gendarussa* (Balaraju et al. 2008 Sudha and Seeni 1994). Balaraju et al. also reported that medium supplemented with IBA enhanced the *in vitro* rooting of *V. agnus-castus* (Balaraju et al. 2008; Sudha and Seeni 1994). In this study, root initiation occurred immediately after the transfer of cultures to the root induction medium without regulators. An efficient rooting protocol to obtain whole plants was established. After 6 weeks of culture, the rooted plantlets were transplanted to a potting mixture, and potted plants were acclimatized for 4 weeks before being transferred to the field. The *ex vitro* survival rate of plantlets was 100%. In 1 year, by using this efficient protocol, 37,600 plants could be produced from a single leaf explant.

Using this method, we can obtain the source of raw materials. Thirteen compounds were isolated from the leaves of micropropagated plants of *H. pogonocalyx*. This is the first report on the chemical investigation of micropropagated *H. pogonocalyx* produced from leaf explants. Most of the popular de-pigmenting agents in current use are toward non-toxic natural products. Reactive oxygen species (ROS) and free radical-mediated reactions are involved in many degenerative and pathological processes, such as neurodegenerative diseases (Lee and Wei 2012). Therefore, these isolated compounds were evaluated for anti-melanogenic activity in human melanocytes and neurocytoprotective activity in PC12 cells in the present study. Among them, acteoside (**7**) exhibited the greatest anti-tyrosinase activity, and luteolin 7-*O*-β-d-glucopyranoside (**11**) exhibited the greatest neurocytoprotective activity. Acteoside is a type of phenylethanoid glycoside found in bitter tea and many medicinal plants, and diverse biological activities (He et al. 2011) including chemopreventive (Hwang et al. 2011), antiallergic (Yamada et al. 2010), and hepatoprotective (Zhao et al. 2009) activities have been reported for this compound. Acteoside was reported to protect cells from oxidative stress and free radical-mediated impairment of endothelial function (Chiou et al. 2004). It was also reported to decrease tyrosinase activity and melanin biosynthesis in B16F10 melanoma cells (Son et al. 2011). However, the anti-tyrosinase activity of acteoside in normal human epidermal melanocytes was confirmed in this study for the first time. The isolated compound, luteolin 7-*O*-β-d-glucopyranoside (**11**), showed the protective effect against 6-OHDA-induced PC12 cells. The result was similar to our previous data (Lin et al. 2009,2012). Rutin (**12**) exhibited protective effect against spatial memory impairment induced by trimethyltin in rats and toxicant-induced hippocampal injury (Koda et al. 2008,2009). In this study, rutin (**12**) showed the neurocytoprotective effect in 6-OHDA-induced NGF-differentiated PC12 cells. These two isolated compounds could be the neurocytoprotective indicators in this plant. Myricetin (**9**) exhibited antiallergic (Shimosaki et al. 2011), antipsychotic-like (Pereira et al. 2011), and anxiolytic (Fernandez et al. 2009) effects. There is no report of neuroprotective activity for myricetin.

## Conclusion

In summary, to our knowledge, this is the first report to develop a method of indirect shoot organogenesis from leaf explants of *H. pogonocalyx*. The method could be developed to supply enough quantity of plant materials for the first chemical and pharmacological investigation. These pharmacological results may promote to development for whitening agents and treatment for neurodegenerative diseases in the future.

## Electronic supplementary material

Additional file 1: Supporting Information. (DOC 106 KB)

Below are the links to the authors’ original submitted files for images.

Authors’ original file for figure 1

Authors’ original file for figure 2

Authors’ original file for figure 3

Authors’ original file for figure 4

Authors’ original file for figure 5

## Abbreviations

BA: 6-benzyladenine
2iP: 6-(γ, γ-dimethylallylamino)-purine
IAA: Indole-3-acetic acid
IBA: Indole-3-butyric acid
NAA: α-naphthalene acetic acid.
