# Supplementary material for: Chemical and pharmacological investigation of micropropagated Hygrophila pogonocalyx produced from leaf explants
Source: Bot Stud. 2013 Oct 30;54:51. doi: 10.1186/1999-3110-54-51 (PMC5430379; doi:10.1186/1999-3110-54-51)
Supplement: Supplementary file 1 — Additional file 1: Supporting Information. (DOC 106 KB) [file 40529_2012_44_MOESM1_ESM.doc]

**Supporting Information**

Luteolin 7-*O*-β-d-glucuronide (**1**)

Yellow powder; UV (MeOH) λ_max_ (log *ε*): 255 (4.2), 268 (4.1), 346 (4.3) nm; ESI-MS *m/z* 461.2 [M-H]^-^; ^1^H-NMR (500 MHz, CD_3_OD): δ 6.73 (1H, s, H-3), 6.78 (1H, d, *J* = 2.0 Hz, H-6), 6.43 (1H, d, *J* = 2.0 Hz, H-8), 7.40 (1H, d, *J* = 2.1 Hz, H-2′), 6.88 (1H, d, *J* = 8.6 Hz, H-5′), 7.43 (1H, dd, *J* = 8.6, 2.1 Hz , H-6′), 5.17 (1H, d, *J* = 7.8 Hz, H-1′′), 3.25 (1H, H-2′′), 3.30 (1H, H-3′′), 3.29 (1H, H-4′′), 3.83 (1H, H-5′′); ^13^C-NMR (125 MHz, CD_3_OD): δ 164.5, 103.1, 181.9, 157.0, 94.5, 162.7, 99.5, 161.1, 105.4, 121.3, 113.5, 145.8, 150.0, 116.0, 119.1, 99.4, 72.9, 76.0, 71.6, 74.7, 171.0.

3-*O*-[β-d-Apiofuranosyl-(1→6)-β-d-glucopyranosyl]oct-1-en-3-ol (**2**)

Amorphous powder; HRESI-MS *m/z* 445.2065 [M+Na]^+^ (calculated for 445.2050); [α]^24^_D_ -63.4 (*c* 3.45, MeOH); ^1^H-NMR (500 MHz, CD_3_OD): δ 5.21 (1H, dd, *J* = 17.2, 2.5 Hz, H-1a), 5.11 (1H, dd, *J* = 10.3, 2.5 Hz, H-1b), 5.86 (1H, ddd, *J* =17.2, 10.3, 7.2 Hz, H-2), 4.09 (1H, dt, *J* = 7.2, 6.6 Hz, H-3), 1.51 (1H, H-4a), 1.67 (1H, H-4b), 1.38 (1H, H-5), 1.29 (1H, H-6), 1.34 (1H, H-7), 0.89 (3H, t, *J* = 13.9, 6.8 Hz, H-8), 4.28 (1H, d, *J* = 8.0 Hz, H-1′), 3.17 (1H, dd, *J* = 9.0, 8.0 Hz, H-2′), 3.31 (1H, H-3′), 3.25 (1H, d, *J* = 9.0 Hz, H-4′), 3.31 (1H, H-5′), 3.56 (1H, H-6′a), 3.93 (1H, dd, *J* = 11.9, 2.2 Hz, H-6′b), 4.98 (1H, d, *J* = 2.3 Hz, H-1′′), 3.87 (1H, d, *J* = 2.3 Hz, H-2′′), 3.94 (1H, d, *J* = 9.7 Hz, H-4′′a), 3.74 (1H, d, *J* = 9.7 Hz, H-4′′b), 3.56 (H-5′′); ^13^C-NMR (125 MHz, CD_3_OD): δ 116.3, 140.8, 83.1, 35.7, 25.7, 33.0, 23.6, 14.4, 103.2, 75.3, 78.2, 71.7, 76.8, 68.4, 110.8, 78.0, 80.6, 75.0, 65.7.

3-*O*-[α-l-Xylopyranosyl-(1→6)-β-d-glucopyranosyl]oct-1-en-3-ol (**3**)

Amorphous powder; ESI-MS *m/z* 421.2 [M-H]^-^; [α]^24^_D_ -47.1 (*c* 3.7，MeOH); ^1^H-NMR (500 MHz, CD_3_OD): δ 5.21 (1H, dd, *J* = 17.3, 1.2 Hz, H-1a), 5.10(1H, dd, *J* = 10.5, 1.2 Hz, H-1b), 5.86 (1H, ddd, *J* = 17.3, 10.5, 6.9 Hz, H-2), 4.12 (1H, dt, *J* = 6.9, 6.6 Hz, H-3), 1.51 (1H, H-4a), 1.66 (1H, H-4b), 1.37 (1H, H-5), 1.28 (1H, H-6), 1.30 (1H, H-7), 0.89 (3H, t, *J* = 13.9, 7.0 Hz, H-8), 4.31 (1H, d, *J* = 7.8 Hz, H-1′), 3.17 (H-2′), 3.30 (H-3′), 3.47 (H-4′), 3.30 (H-5′), 3.73 (1H, d, *J* = 11.5 Hz, H-6′a), 4.01 (1H, dd, *J* = 11.5, 1.9 Hz, H-6′b), 4.33 (1H, d, *J* = 7.3 Hz, H-1′′), 3.17 (H-2′′), 3.32 (H-3′′), 3.30 (H-4′′), 3.17 (H-5′′a), 3.84 (1H, dd, *J* = 11.5, 5.3 Hz, H-5′′); ^13^C-NMR (125 MHz, CD_3_OD): δ 116.2, 140.9, 82.7, 35.7, 25.6, 33.0, 23.6, 14.4, 103.3, 75.3, 78.1, 71.2, 77.6, 69.5, 105.3, 74.8, 77.0, 71.5, 66.8.

β-Sitosterol (**4**) and stigmasterol (**5**)

Amorphous powder; [α]^24^_D_ -47.1 (*c* 3.7, *n*-Hexane); ^1^H-NMR (500 MHz, CD_3_OD): δ 3.52 (1H, H-3), 5.35 (1H, H-6), 0.68 (3H, s, Me-18 (**5)**), 0.70 (3H, s, Me-18 (**4)**), 1.01 (3H, s, Me-19), 0.92 (3H, d, *J* = 6.4 Hz, Me-21 (**5**)), 1.03 (3H, d, *J* = 8.8 Hz, Me-21 (**4**)), 5.15 (1H, dd, *J* = 15.2, 8.6 Hz, H-22 (**4**)), 5.02 (1H, dd, *J* =15.2, 8.6 Hz H-23 (**4**)); ^13^C-NMR (125 MHz, CD_3_OD): δ 37.3, 31.7, 71.8, 42.3, 140.8, 121.7, 31.9, 31.9, 50.2, 36.5, 21.1, 40.5, 39.7, 42.3, 42.2, 56.9, 24.4, 24.3, 28.9, 29.7, 56.0, 55.9, 12.0, 12.2, 19.4, 36.5, 40.5, 19.0, 21.2, 32.0, 138.3, 25.4, 129.3, 42.3, 51.2, 29.7, 31.9, 19.4, 21.1, 19.0, 24.3, 25.4, 12.0, 12.2.

β-Ethoxylacteoside (**6**)

Amorphous powder; HRESI-MS *m/z* 691.2231 [M + Na]^+^ (calculated for 691.2214); [α]^24^_D_ -47.1 (*c* 1.95, MeOH); UV (MeOH) λ_max_ (log *ε*): 221 (4.22), 332 (4.22) nm; ^1^H-NMR (500 MHz, CD_3_OD): δ 7.04 (1H, d, *J* = 1.7 Hz, H-2), 6.76 (1H, d, *J* = 8.2 Hz, H-5), 6.94 (1H, d, *J* = 8.2, 1.7 Hz, H-6), 7.58 (1H, d, *J* = 15.9 Hz, H-7), 6.26 (1H, d, *J* = 15.9 Hz, H-8), 6.77 (1H, d, *J* = 1.9 Hz, H-2′), 6.77 (1H, d, *J* = 8.1 Hz, H-5′), 6.65 (1H, dd, *J* = 8.1, 1.9 Hz, H-6′), 3.83 (1H, dd, *J* = 10.9, 2.9 Hz, H-α_a_), 3.71 (1H, dd, *J* = 10.9, 8.8 Hz, H-α_b_), 4.46 (1H, dd, *J* = 8.8, 2.9 Hz, H-β), 3.44 (2H, q, *J* = 7.0 Hz, H-1′′), 1.16 (3H, t, *J* = 7.0 Hz, H-2′′), 4.43 (1H, d, *J* = 7.9 Hz, H-1′′′), 3.71 (1H, H-2′′), 3.83 (1H, H-3′′), 4.91 (1H, H-4′′), 3.42 (1H, H-5′′), 3.51 (1H, 1H, H-6′′a), 3.60 (1H, d, *J* = 10.2 Hz, H-6′′b), 5.21 (1H, s, H-1′′′′), 3.91 (1H, H-2′′′′), 3.63 (1H, H-3′′′′), 3.28 (1H, H-4′′′′), 3.58 (1H, H-5′′′′), 1.09 (3H, d, *J* = 6.1 Hz, H-6′′′′); ^13^C-NMR (125 MHz, CD_3_OD): δ 127.5, 115.1, 146.9, 150, 116.5, 123.3, 148.1, 114.6, 168.3, 131.5, 114.9, 146.6, 146.4, 116.3, 119.7, 75.2, 82.6, 65.1, 15.5, 104.6, 76.1, 81.5, 70.5, 76.4, 62.4, 103.0, 72.4, 71.4, 73.8, 72.1, 18.4.

Acteoside (**7**)

Amorphous powder; ESI-MS *m/z* 623.3 [M-H]^-^; [α]^24^_D_ -83.1 (*c* 6.95, MeOH); UV (MeOH) λ_max_ (log *ε*): 243 (4.4), 305 (4.5), 332 (4.7) nm; ^1^H-NMR (500 MHz, CD_3_OD): δ 7.05 (1H, brs, H-2), 6.77 (1H, d, *J* = 7.9 Hz, H-5), 6.95 (1H, d, *J* = 7.9 Hz, H-6), 7.59 (1H, d, *J* = 15.9 Hz, H-7), 6.27 (1H, d, *J* = 15.9 Hz, H-8), 6.68 (1H, s, H-2′), 6.67 (1H, d, *J* = 7.0 Hz, H-5′), 6.56 (1H, d, *J* = 7.0 Hz, H-6′), 4.03 (1H, dd, *J* = 16.6, 7.7 Hz, H-αa), 3.70 (1H, dd, *J* = 16.6, 7.7 Hz, H-α_b_), 2.79 (1H, H-β), 4.37 (1H, d, *J* = 7.6 Hz, H-1′′), 3.51 (1H, H-2′′), 3.81 (1H, H-3′′), 4.91 (1H, H-4′′), 3.39 (1H, H-5′′), 3.54 (1H, H-6′′a), 3.62 (1H, H-6′′b), 5.18 (1H, s, H-1′′′), 3.53 (1H, H-2′′′), 3.92 (1H, H-3′′′), 3.31 (1H, H-4′′′), 3.51 (1H, H-5′′′), 1.09 (3H, d, *J* = 6.1 Hz, H-6′′′); ^13^C-NMR (125 MHz, CD_3_OD): δ 127.6, 115.2, 146.8, 149.7, 116.5, 123.2, 148.0, 114.7, 168.0, 131.5, 117.1, 146.1, 144.6, 116.3, 121.0, 72.3, 36.5, 104.1, 76.0, 81.6, 70.4, 76.2, 62.3, 103.0, 72.0, 72.2, 73.8, 70.6, 18.4.

Isoacteoside (**8**)

Amorphous powder; ESI-MS *m/z* 623.33 [M-H]^-^; [α]^24^_D_ -39.5 (*c* 2.40, MeOH); UV (MeOH)λmax (log *ε*): 219 (4.2), 329 (4.2) nm; ^1^H-NMR (500 MHz, CD_3_OD): δ 7.03 (1H, d, *J* = 1.6 Hz, H-2), 6.76 (1H, d, *J* = 8.2 Hz, H-5), 6.88 (1H, dd, *J* = 8.2, 1.6 Hz, H-6), 7.55 (1H, d, *J* = 15.9 Hz, H-7), 6.28 (1H, d, *J* = 15.9 Hz, H-8), 6.66 (1H, d, *J* = 1.7 Hz, H-2′), 6.63 (1H, d, *J* = 8.2 Hz, H-5′), 6.52 (1H, dd, *J* = 8.2, 1.7 Hz, H-6′), 3.94 (1H, H-α_a_), 3.70 (1H, H-α_b_), 2.77 (2H, t, *J* = 6.4 Hz, H-β), 4.32 (1H, d, *J* = 7.8 Hz, H-1′′), 3.30 (1H, H-2′′), 3.51 (1H, H-3′′), 3.40 (1H, H-4′′), 3.70 (1H, H-5′′), 4.48 (1H, dd, *J* = 1.7 Hz, 11.0 Hz, H-6′′a), 4.36 (1H, dd, *J* = 5.8, 11.0 Hz, H-6′′b), 5.17 (1H, s, H-1′′′), 3.70 (1H, H-2′′′), 3.52 (1H, H-3′′′), 3.40 (1H, H-4′′′), 3.98 (1H, H-5′′′), 1.24 (3H, d, *J* = 6.2 Hz, H-6′′′); ^13^C-NMR (125 MHz, CD_3_OD): δ 127.6, 115.1, 146.8, 149.7, 116.5, 123.2, 147.3, 114.8, 168.1, 131.4, 117.1, 146.1, 144.7, 116.4, 121.3, 72.3, 36.7, 104.4, 75.7, 84.0, 70.4, 72.3, 64.6, 102.7, 72.4, 75.4, 74.0, 70.0, 17.9.

Myricitrin (**9**)

Yellow powder; UV (MeOH) λ_max_ (log *ε*): 261 (4.3), 286 (4.5), 305 (4.1) nm; [α]^27^_D_ -137.4 (*c* 0.5, MeOH); ^1^H-NMR (500 MHz, CD_3_OD): δ 6.17 (1H, d, *J* = 1.7 Hz, H-6), 6.33 (1H, d, *J* = 1.7 Hz, H-8), 6.94 (1H, s, H-2′), 5.31 (1H, brs, H-1′′), 4.21 (1H, H-2′′), 3.78 (1H, dd, *J* = 10.4, 2.9 Hz, H-3′′), 3.56 (1H, t, *J* = 10.2 Hz, H-4′′), 3.63 (1H, H-5′′), 0.95 (1H, d, *J* = 6.2 Hz, H-6′′); ^13^C-NMR (125 MHz, CD_3_OD): δ 158.6, 136.3, 179.6, 163.2, 100,1, 163.2, 94.9, 159.4, 105.6, 121.9, 109.6, 146.9, 138.0, 146.9, 109.6, 103.6, 71.9, 72.1,73.4, 72.0, 17.7.

Quercetin (**10**)

Yellow powder; UV (MeOH) λ_max_ (log *ε*): 256 (4.1), 290 (4.3), 376 (4.2) nm; ^1^H-NMR (500 MHz, CD_3_OD): δ 6.17 (1H, d, *J* = 2.2 Hz, H-6), 6.38 (1H, d, *J* = 2.2 Hz, H-8), 7.72 (1H, d, *J* = 2.2 Hz, H-2′), 6.87 (1H, d, *J* = 8.5 Hz, H-5′), 7.62 (1H, dd, *J* = 8.5, 2.3 Hz , H-6′)

Luteolin 7-*O*-β-d-glucopyranoside (**11**)

Yellow powder; ESI-MS *m/z* 447.2 [M - H]^-^; UV (MeOH) λ_max_ (log *ε*): 253 (4.1), 266 (4.0), 347 (4.2) nm; ^1^H-NMR (500 MHz, CD_3_OD): δ 6.74 (1H, s, H-3), 6.78 (1H, d, *J* = 2.1 Hz, H-6), 6.43 (1H, d, *J* = 2.1 Hz, H-8), 7.41 (1H, d, *J* = 2.2 Hz, H-2′), 6.89 (1H, d, *J* = 8.4 Hz, H-5′), 7.44 (1H, dd, *J* = 8.4, 2.2 Hz, H-6′), 5.07 (1H, d, *J* = 7.5 Hz, H-1′′), 3.24 (1H, H-2′′), 3.29 (1H, H-3′′), 3.17 (1H, H-4′′), 3.43 (1H, H-5′′), 3.47 (1H, dd, *J* = 11.5, 5.8 Hz, H-6′′a), 3.70 (1H, d, *J* = 11.5 Hz, H-6′′b); ^13^C-NMR (125 MHz, CD_3_OD): δ 164.5, 103.1, 181.9, 156.9, 94.7, 162.9, 99.5, 161.1, 105.4, 121.2, 113.5, 145.8, 150.2, 116.0, 119.2, 99.9, 73.1, 76.4, 69.5, 77.2, 60.6.

Rutin (**12**)

Yellow powder; UV (MeOH) λ_max_ (log *ε*): 257 (4.1), 357 (4.3) nm; [α]^24^_D_  -10.1°(*c* 0.95, MeOH); ^1^H-NMR (500 MHz, CD_3_OD): δ 6.20 (1H, d, *J* = 1.1 Hz, H-6), 6.39 (1H, d, *J* = 1.1 Hz, H-8), 7.66 (1H, d, *J* = 1.9 Hz, H-2′), 6.87 (1H, d, *J* = 8.4 Hz, H-5′), 7.62 (1H, dd, *J* = 8.4, 1.9 Hz, H-6′), 5.10 (1H, d, *J* = 7.6 Hz, H-1′′), 3.46 (1H, H-2′′), 3.41 (1H, H-3′′), 3.26 (1H, H-4′′), 3.31 (1H, H-5′′), 3.38 (1H, H-6′′a), 3.80 (1H, d, *J* = 10.2 Hz, H-6′′b), 4.51 (1H, d, *J* = 1.5 Hz, H-1′′′), 3.62 (1H, *J* = 1.5 Hz, H-2′′′), 3.52 (1H, H-3′′′), 3.27 (1H, H-4′′′), 3.45 (1H, H-5′′′), 1.11 (3H, d, *J* = 6.2 Hz, H-6′′′); ^13^C-NMR (125 MHz, CD_3_OD): δ 158.5, 135.6, 179.4 , 163.0, 100.0, 166.2, 94.9, 159.3, 105.6, 123.1, 117.7, 145.9, 149.8, 116.1, 123.6, 104.7, 75.7, 78.2, 71.4, 77.2, 68.6, 102.4, 72.1, 72.2, 73.9, 69.7, 17.9.

Isoquercitrin (**13**)

Yellow powder; UV (MeOH) λ_max_ (log *ε*): 261 (4.1), 268 (4.5), 365 (4.3) nm; [α]^27^_D_  -3.4 (*c* 0.5, MeOH); ^1^H-NMR (500 MHz, CD_3_OD): δ 6.18 (1H, brs, H-6), 6.37 (1H, brs, H-8), 7.70 (1H, brs, H-2′), 6.86 (1H, d, *J* = 7.1 Hz, H-5′), 7.57 (1H, d, *J* = 7.1 Hz , H-6′), 5.23 (1H, d, *J* = 7.3 Hz, H-1′′), 3.47 (1H, H-2′′), 3.23 (1H, H-3′′), 3.33 (1H, H-4′′), 3.42 (1H, H-5′′)，3.57 (1H, dd, *J* = 11.8, 5.1 Hz, H-6′′a), 3.70 (1H, d, *J* = 11.8 Hz, H-6′′b); ^13^C-NMR (125 MHz, CD_3_OD): δ 159.0, 135.6 179.4, 163.0, 100.0, 166.3, 94.8, 158.5, 105.6, 123.2, 123.1, 145.9, 149.9, 116.0, 123.1, 104.4, 75.7, 78.4, 71.2, 78.1, 62.6.
