# Supplementary figures and images for: Chemical and pharmacological investigation of micropropagated Hygrophila pogonocalyx produced from leaf explants
Source: Bot Stud. 2013 Oct 30;54:51. doi: 10.1186/1999-3110-54-51 (PMC5430379; doi:10.1186/1999-3110-54-51)

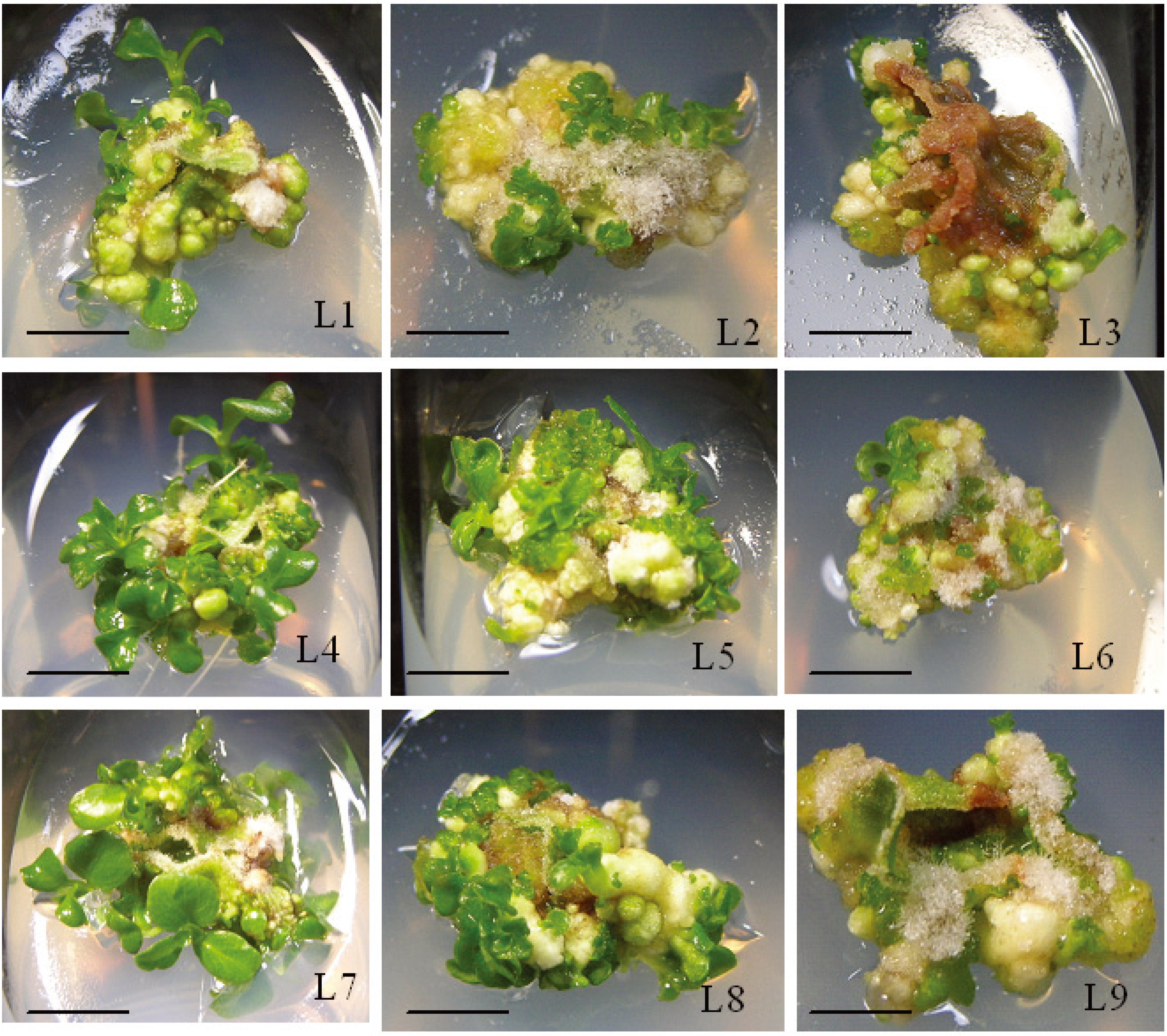

Supplement: Supplementary file 2 — Authors’ original file for figure 1 [file 40529_2012_44_MOESM2_ESM.tiff]

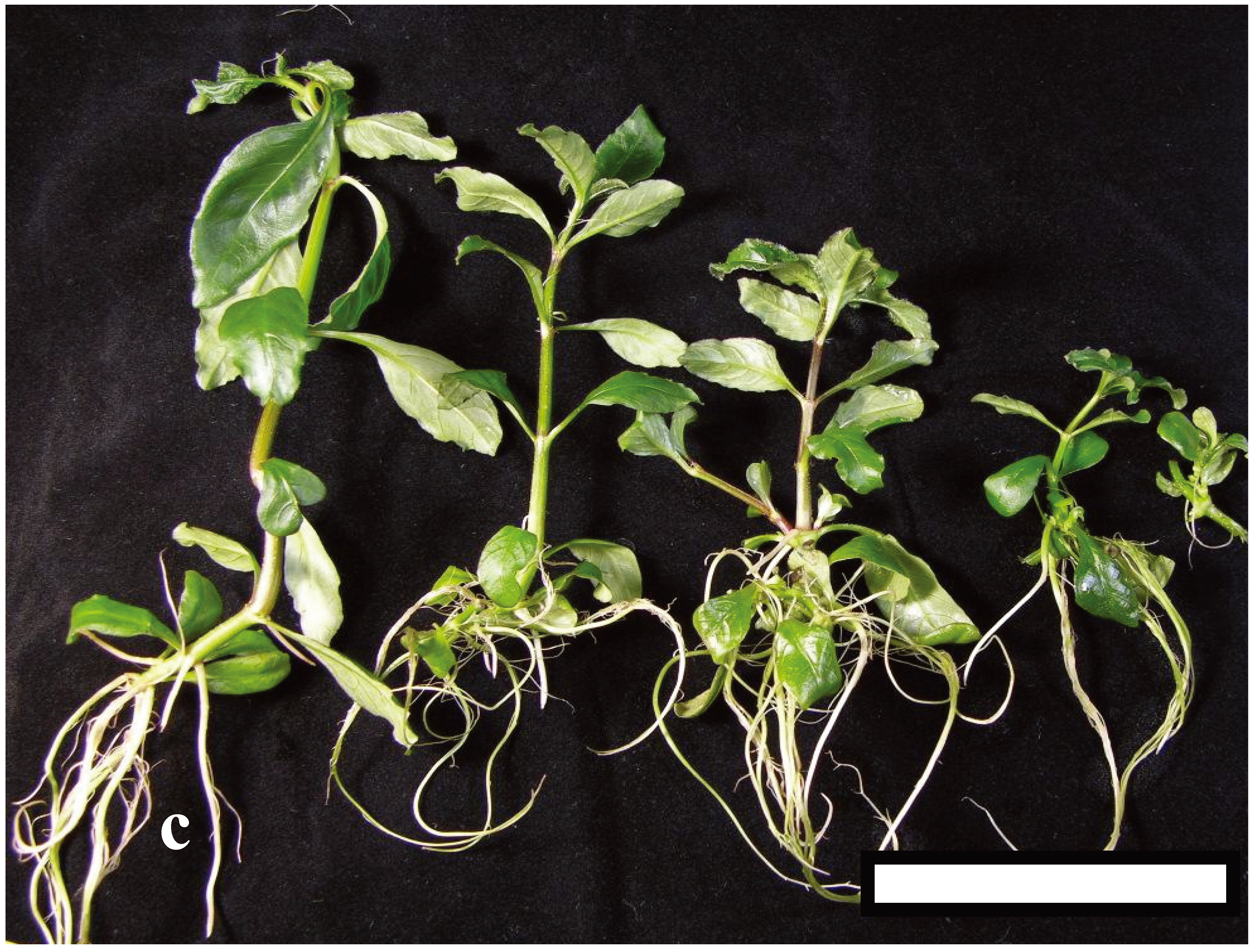

Supplement: Supplementary file 3 — Authors’ original file for figure 2 [file 40529_2012_44_MOESM3_ESM.tiff]

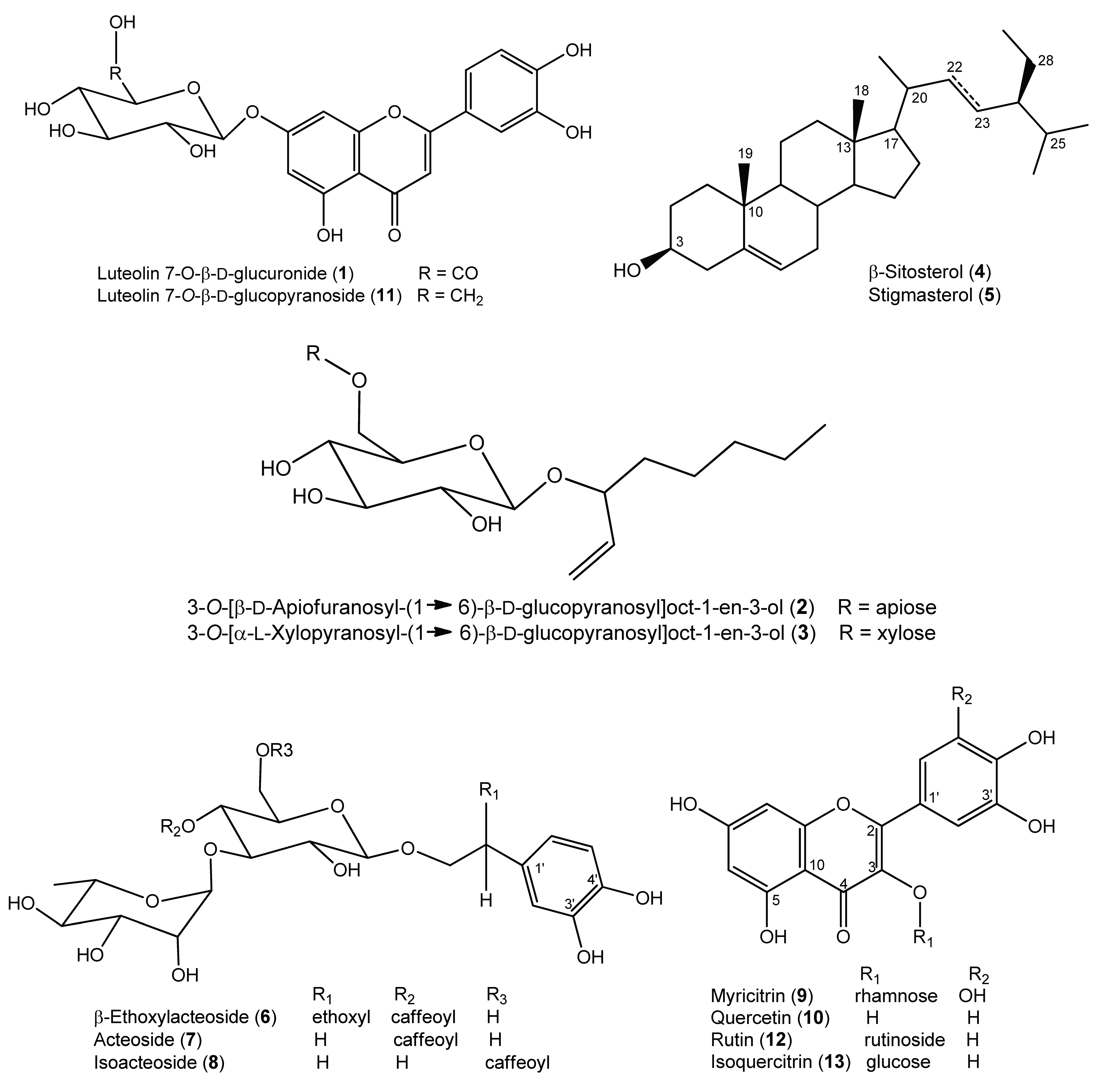

Supplement: Supplementary file 4 — Authors’ original file for figure 3 [file 40529_2012_44_MOESM4_ESM.tiff]
